# Beyond Branching: Unlocking the Catalytic Versatility of Branching Enzymes for the Design of Diverse α-Glucan Structures

**DOI:** 10.3390/molecules31162821

**Published:** 2026-08-13

**Authors:** Maurice K. H. Essers, Hans Leemhuis, Johannes H. Bitter, Lambertus A. M. van den Broek

**Affiliations:** 1 Wageningen Food & Biobased Research, P.O. Box 17, 6700 AA Wageningen, The Netherlands; ben.vandenbroek@wur.nl; 2 Royal Avebe, Avebe Innovation Center, Zernikelaan 8, 9747 AA Groningen, The Netherlands; hans.leemhuis@avebe.com; 3 Biobased Chemistry and Technology, AFSG, Wageningen University, Bornse Weilanden 9, 6708 WG Wageningen, The Netherlands; harry.bitter@wur.nl

**Keywords:** chemically modified starches, interlinked catalytic activities, synergistic enzymatic functions, primary and secondary reactions, digestibility, enzymatic restructuring of starch, personal and home care applications

## Abstract

Starch-modifying glycoside hydrolases (GHs) typically operate via a retaining double-displacement mechanism, involving formation of a covalent glycosyl–enzyme intermediate. This intermediate can be resolved either by water, resulting in hydrolysis, or by a glucan acceptor, leading to transglucosylation. Many GHs exhibit both catalytic activities, although they are classified according to their predominant reaction. For example, branching enzymes (BEs) catalyse α-(1→4) bond cleavage and α-(1→6) branch formation via transglucosylation, while also exhibiting minor hydrolytic and disproportionation activities that broaden their catalytic repertoire. More recent research indicates that the different catalytic activities of BEs can be interconnected, thereby collectively determining the final α-glucan architecture. This challenges the classical view that the predominant branching activity of BEs is catalysed independently. Moreover, the balance between these coupled activities influences substrate specificity and can broaden the substrate scope to include chemically modified starches. Furthermore, the co-application of BEs with other GHs reveals synergistic interactions between catalytic activities, enabling the generation of α-glucan structures that cannot be produced by any of the enzymes individually. In this perspective paper, and based on recent developments, we argue that the catalytic framework of BEs provides multiple strategies for tailoring diverse α-glucan architectures. This enables modulation of structural features across hierarchical levels, from supramolecular to macromolecular organisation. As a result, BEs represent versatile tools for engineering starch functionality beyond digestibility, extending their potential toward pharmaceutical and non-food applications. Looking ahead, we discuss how enzyme-designed and chemically functionalised α-glucan polymers may emerge as a new class of sustainable materials. These materials could provide biodegradable, water-soluble, and renewable alternatives to petrochemical-derived polymers used in personal and home care products.

## 1. Introduction

In the late nineteenth and early twentieth centuries, starch-active enzymes were almost exclusively viewed as hydrolase, particularly α- and β-amylases used in brewing and malting for fermentable sugar production. This view shifted in the mid-20th century when unexpected amylase reaction products revealed activities beyond simple hydrolase. A key breakthrough was the discovery of cyclodextrin glucanotransferase (CGTase) in the 1950s, demonstrating intramolecular transglucosylation of α-(1→4)-glucans to form cyclic products [1].

Over time, a broader range of transferase activities has been identified. These include glycogen branching enzymes (BEs), which introduce α-(1→6)-linked branches, and 4-α-glucanotransferases (4αGT, amylomaltases), which catalyse α-(1→4) glucan transfer [2,3,4,5].

Between 1991 and 1993, Henrissat and co-workers [6] classified glycoside hydrolases (GHs), including transglucosylation-active enzymes, into families based on sequence similarity and evolutionary relationships. This classification was incorporated into the CAZy database (launched in 1998, http://www.cazy.org) [7], which organizes carbohydrate-active enzymes according to conserved sequence, structural, and mechanistic features. GH families can be further divided into subfamilies to reflect functional diversity. Within CAZy, CGTases are classified as GH13_20, BEs as GH13_8/GH13_9 and GH57_5, and amylomaltases (4-α-glucanotransferases) as GH77_1.

More recently, GH70 enzymes with novel activities, such as 4,6-α-glucanotransferases and enzymes forming α-(1→3) linkages, have been described, further expanding the diversity of α-glucan structures that can be generated [8,9,10,11,12,13].

**Figure 1:**
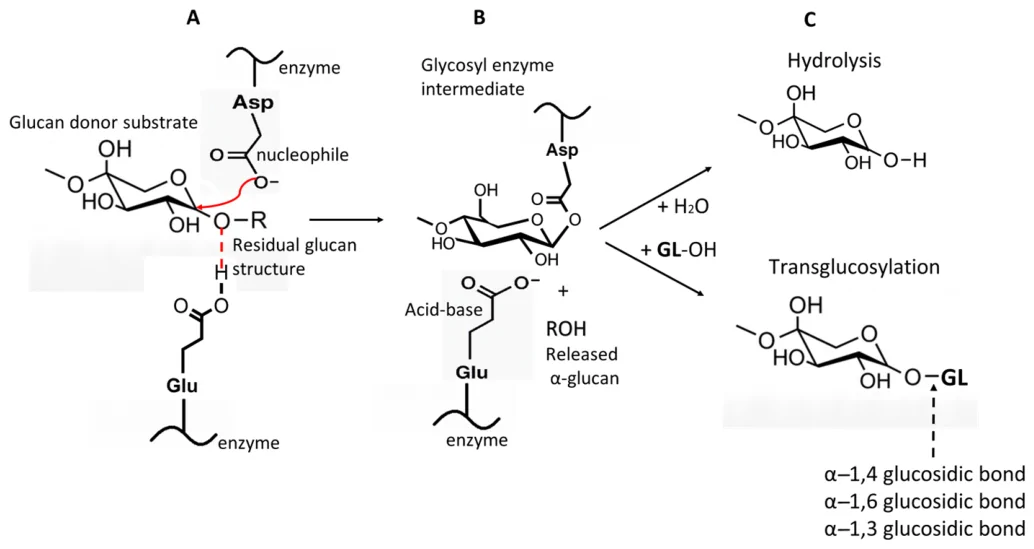
Schematic representation of the double-displacement mechanism of glycoside hydrolases. (**A**) Formation of the Michaelis complex; the red arrow indicates nucleophilic attack on the anomeric carbon, and the red dotted line indicates protonation of the glycosidic oxygen. (**B**) Formation of the covalent glucosyl–enzyme intermediate. (**C**) Deglucosylation via hydrolysis (+H_2_O) or transglucosylation (+GL–OH). Glu, glutamic acid; Asp, aspartic acid; GL–OH, α-glucan acceptor.

Despite their different functions, all these GH families use the same retaining double-displacement mechanism. The reaction starts when a nucleophile in the enzyme attacks the anomeric carbon of the donor substrate, while a second catalytic residue protonates the glucosidic oxygen. This forms a covalent glucosyl–enzyme intermediate. In the next step, this intermediate is broken down either by water, producing hydrolysis products, or by a carbohydrate acceptor, producing transglucosylation products (Figure 1) [14,15,16].

Thus, hydrolysis and transglucosylation represent alternative outcomes of the same catalytic mechanism, depending on whether water or a carbohydrate acceptor reacts with the glycosyl–enzyme intermediate [17]. This outcome is largely dictated by enzyme architecture: open, solvent-exposed active sites with flexible loops favour hydrolysis, whereas more enclosed active sites that restrict water access and precisely position acceptor glucan substrates promote transglucosylation [18,19,20,21]. The specific reaction type, branching, disproportionation, or cyclisation, is further determined by how the acceptor glucan is oriented within the catalytic subsites, governing the formation of α-(1→4) or α-(1→6) linkages and whether transfer occurs intra- or intermolecularly [3,22,23].

Although GHs within the same family share conserved catalytic mechanisms and family-specific protein folds, their catalytic outcomes can vary considerably [6]. For example, the GH13 family comprises more than 35 subfamilies, including enzymes that catalyse hydrolysis (α-amylases), branching reactions (BEs), and cyclisation reactions (CGTases) [24].

Intriguingly, the same transglucosylation reactions can arise in structurally distinct GH families with different protein folds, a phenomenon consistent with convergent evolution. For instance, α-amylase and branching activities are found in both GH13 (canonical (β/α)_8_ barrel) and GH57 (β/α)_7_ barrel), while disproportionation activities occur in GH13, GH57, and GH77 ((β/α)_8_ barrel) [25,26,27,28,29]. This highlights that sequence-based CAZy family classification alone is insufficient to reliably predict enzyme function.

Enzyme discovery efforts continue to expand the GH families containing BEs, particularly GH13 and GH57, which together comprise thousands of predicted sequences [22]. Despite this vast sequence diversity, to date only approximately 30–45 BEs have been experimentally characterized, with GH13 exhibiting especially high structural and functional diversity (Table 1).

Initial studies on BEs, dating back to the early 1980s [54], focused primarily on substrate specificity and optimisation of the primary (dominant) branching reaction, largely in the context of food applications and modulation of human starch digestibility. In contrast, secondary reaction pathways occurring during enzymatic branching reactions, such as minor hydrolytic activities, as well as their broader industrial applications, have received comparatively limited attention.

More recent studies (since ~2020) adopt a more integrated perspective in which primary and secondary reactions are treated as interlinked activities in determining the final glucan structure [27,55]. The substrate specificity of BEs has also been recently extended to chemically modified starches [56], indicating tolerance towards introduced functional groups. Furthermore, recent studies show synergistic effects when combining different BEs or pairing BE with 4αGT, enabling α-glucan structures that are not possible with single enzymes [57]. These findings highlight the versatile role of BEs in shaping α-glucan architectures.

Increasingly, biocatalysis is shifting from enzyme discovery toward the rational design of enzyme-generated molecular architectures and their associated structure–function relationships [38,56,57,58,59]. While BEs have traditionally been exploited to modulate starch for human digestibility, particularly by retarding glucose release, a deeper understanding of their catalytic repertoire and expanded substrate scope offers opportunities to extend their application far beyond the nutritional domain. Building on recent advances in the mechanistic understanding and engineering of BE-mediated α-glucan structures, these insights provide a foundation for the development of novel starch-based materials and functionalities across diverse industrial sectors.

In this perspective paper we examine how these advances in catalytic understanding can be leveraged for the targeted engineering of α-glucan structures with defined static (structural) and dynamic (functional, time-dependent) properties. We further highlight two key application areas: (i) healthier food systems with improved texture and controlled digestibility, and (ii) biodegradable, water-soluble polymers based on tailored α-glucan architectures derived from BE-modified, chemically substituted starches. These offer sustainable alternatives to petrochemical-based polymers used in personal and home care products.

## 2. BE’s Primary and Secondary Reactions Influencing Starch Restructuring

Structural changes induced by BE modification of starch are driven by transglucosylation and secondary reactions [60]. These changes include an increased α-1,6/α-1,4 glucosidic bond ratio, as well as altered Mw and branch chain-length distribution. These features can be tuned by the BE source and type, kinetic control of primary (predominant) and secondary (alternative) activities, co-application with other enzymes, and substrate specificity.

### 2.1. Impact of the Reaction Pattern on Final Glucan Architecture

**Figure 2:**
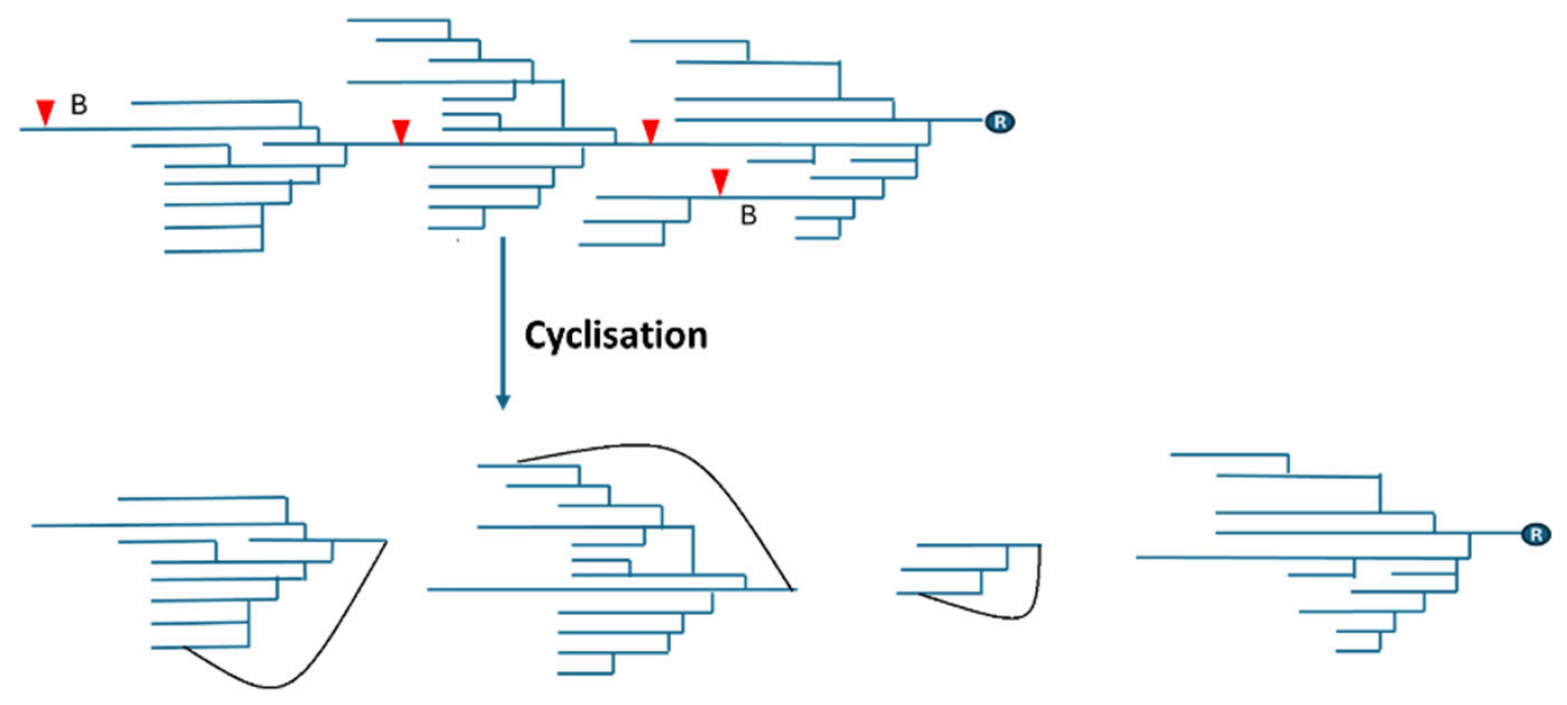
Intramolecular cyclisation reaction. The blue line represents the amylopectin structure; R indicates reducing end groups; B denotes internal chains connecting amylopectin clusters; red triangles indicate glycosidic cleavage sites; and the black line represents the intramolecular transglucosylated branch formed during cyclisation [65].

Starch BEs (EC 2.4.1.18, GH13) catalyse α-(1→6) branch formation by cleaving α-(1→4) bonds and transferring glucan segments to the C6 hydroxyl group of glucose residues [61]. This transfer can occur inter- or intramolecularly. Intramolecular transfer results in cyclisation, producing cyclic glucans and reducing Mw without forming new reducing ends [62,63]. This intramolecular activity has been reported for bacterial BEs, including those from *Bacillus stearothermophilus* and *Bacillus cereus* [62,64,65], as illustrated in Figure 2.

**Figure 3:**
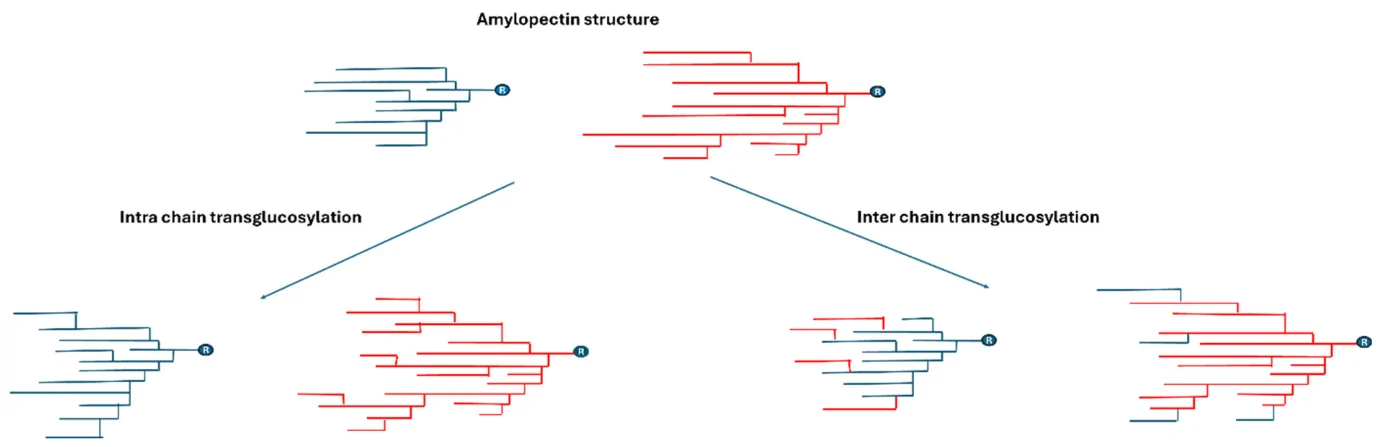
Interchain and intrachain transglucosylation reactions catalysed by branching enzymes (BEs). R denotes reducing end groups, while the blue and red lines represent individual amylopectin molecules.

To date, only a limited number of BEs have been reported to exhibit cyclisation activity, including GH13 branching enzymes from *Bacillus stearothermophilus* and *Bacillus cereus*, which perform intramolecular glucan transfer through alternative acceptor positioning to generate cyclic α-glucans [62,64,65,66]. For most, reactions are described as inter- or intramolecular transglucosylation (Figure 3), although these mechanisms are difficult to distinguish based on final glucan structures from native starch and are rarely conclusive.

Nevertheless, recent studies suggest a stronger influence of reaction mode on the resulting glucan architecture than previously assumed. For example, *Rhodothermus obamensis* branching enzyme (RoBE) has been shown to predominantly catalyse intermolecular transglucosylation [56], enabling the exchange of structural features between distinct starch polymers and generating architectures not achievable via intramolecular transfer alone.

Collectively, these reaction modes increase α-glucan structural diversity but differ in the resulting molecular architectures [67]. Although inter- and intramolecular transglucosylation are not expected, in principle, to decrease Mw, a reduction is often observed experimentally. This may result from cyclisation reactions but is more likely due to secondary hydrolytic activity causing glucan chain cleavage, as cyclisation activity has only been reported for a limited number of BEs [64,65,66].

### 2.2. Secondary Hydrolytic Activity of BEs

Earlier studies commonly reported starch Mw reduction by BEs based solely on the final glucan Mw, without distinguishing whether this resulted from cyclisation or hydrolysis [31,32,40]. Subsequent work indicated the involvement of hydrolytic side activity, as evidenced by an increase in reducing end groups [50].

This hydrolytic activity has been observed in both GH13 and GH57 BEs and contributes to Mw reduction during catalysis. GH57 enzymes often show relatively higher hydrolytic activity. The GH57 BE from *Thermotoga maritima* MSB8 was initially identified as an intracellular α-amylase in 2006 [68]. Subsequent studies revealed that the AmyC amino acid sequence contains characteristic GH57 motifs and exhibits higher branching activity than hydrolytic activity [69]. Hydrolytic activity among GH57 BEs was further reported for enzymes from *Thermococcus kodakarensis*, *Thermococcus litoralis*, and *Thermococcus hydrothermalis*, which produced lower-Mw glucans without a corresponding increase in branching frequency [25,26,53,70,71].

Among GH13 enzymes, thermophilic BEs from *Rhodothermus marinus*, *Petrotoga mobilis*, and *Butyrivibrio fibrisolvens* display measurable α-(1→4)-hydrolytic activity upon prolonged incubation with starch polymers, including amylopectin and amylose [25,35].

This behaviour reflects the multifunctional catalytic nature of BEs and highlights the significant impact of hydrolytic side activity on the resulting glucan structure, including Mw.

Earlier studies [50] largely regarded this hydrolytic activity as a separate, stochastic side reaction arising from the same catalytic mechanism. However, more recent work [72] suggests that it plays an integrated and functionally relevant role in shaping glucan architecture by enzymatically remodelling high-Mw starch substrates.

### 2.3. Interconnected Hydrolysis and Branching Activities

Recent studies employing the BE (RoBE) from *R*. *obamenis* have demonstrated that hydrolysis-driven Mw reduction of high-Mw starch occurs during the early stages of the reaction, generating a dominant high-Mw fraction (>100 kDa; >90% *w*/*w*) alongside a minor population of low-Mw linear glucan fractions attributed to hydrolytic cleavage. The narrow Mw distribution is attributed to internal (endo-type) hydrolysis, which selectively cleaves interconnecting B-chains that link clusters within the amylopectin structure, while preserving external branches and maintaining sufficient chain length for subsequent reactions [72].

**Figure 4:**
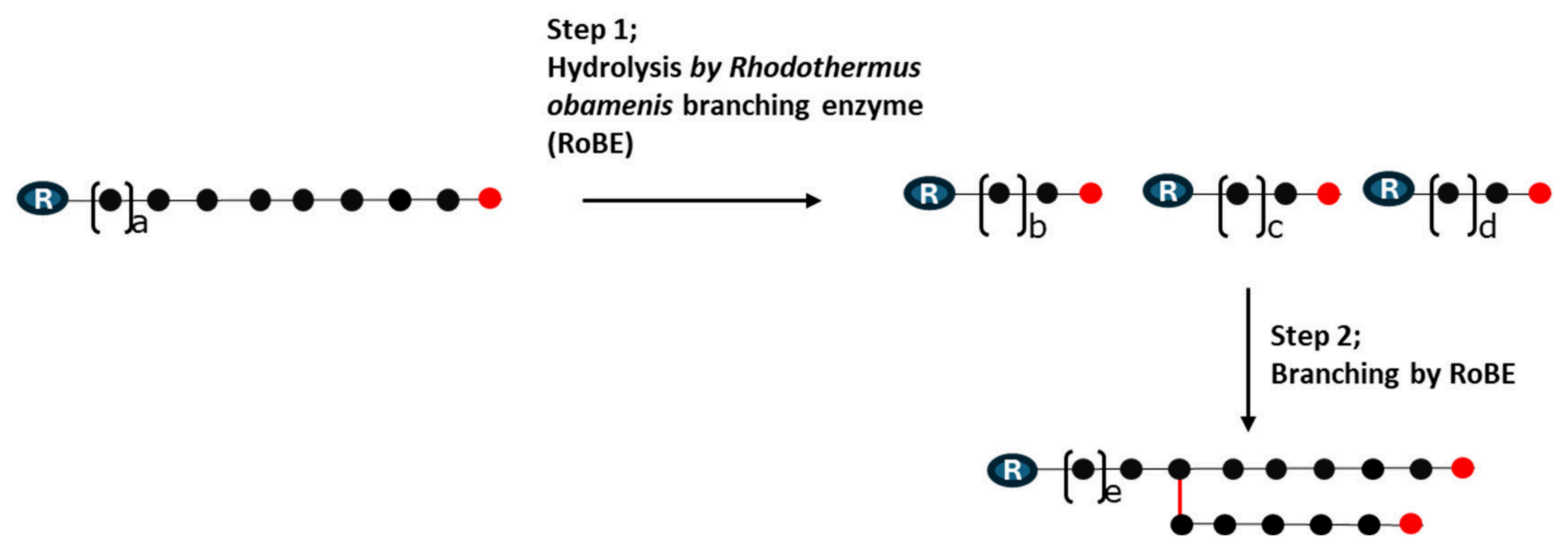
Two-step mechanism of hydrolysis followed by branching catalysed by the *Rhodothermus obamensis* branching enzyme. Blue dots represent reducing ends, while red dots indicate non-reducing ends. The red line represents the newly formed α-1,6-glucosidic bond generated during the branching step. Black dots represent glycosidic moieties within the glucan backbone. Labels a–e denote different levels of degree of polymerisation of the α-glucan chain [72].

Once a critical Mw is reached, donor and acceptor chains can be simultaneously accommodated in the active site, allowing competition with water and initiating branching. This indicates a transition from endo-acting, hydrolysis-dominated catalysis to externally driven transglucosylation within the same catalytic mechanism. Accordingly, hydrolysis serves as a prerequisite for branching and should be viewed as an integral part of starch restructuring rather than a side reaction (Figure 4).

Overall, hydrolysis and branching appear to be temporally separated but mechanistically coupled processes within the same catalytic system. RoBE’s dual activity can be exploited to modulate glucan structure by adjusting branching density while maintaining an approximately constant Mw. This enables control at both the molecular and supramolecular levels, as Mw and the α-1,6/α-1,4 linkage ratio determine hydrodynamic volume and intrinsic viscosity.

In addition, starch-active transferases such as 4αGT and CGTases [1,28,73] catalyse chain elongation, cyclisation, and other transglucosylation reactions. Similarly, BEs also exhibit secondary transferase activities in addition to hydrolytic side activities.

### 2.4. α-1,4-Transglucosylation Activity as a Precursor of Branching Enzyme Function

BEs can also catalyse disproportionation reactions, in which a glucan fragment is transferred to the C4 hydroxyl group of another glucan chain instead of forming an α-(1→6) branch [27,74]. This activity has been observed in several GH13 glycogen BEs, including those from *Rhodothermus*, *Bacillus*, and *Butyrivibrio* spp. GH57 BEs likewise exhibit α-(1→4) transglucosylation activity. Enzymes from *T*. *kodakarensis*, and *Thermotoga maritima* have been shown to produce glucan chains longer than the initial substrate, providing direct evidence for disproportionation activity in GH57 glycogen BEs [60].

**Figure 5:**
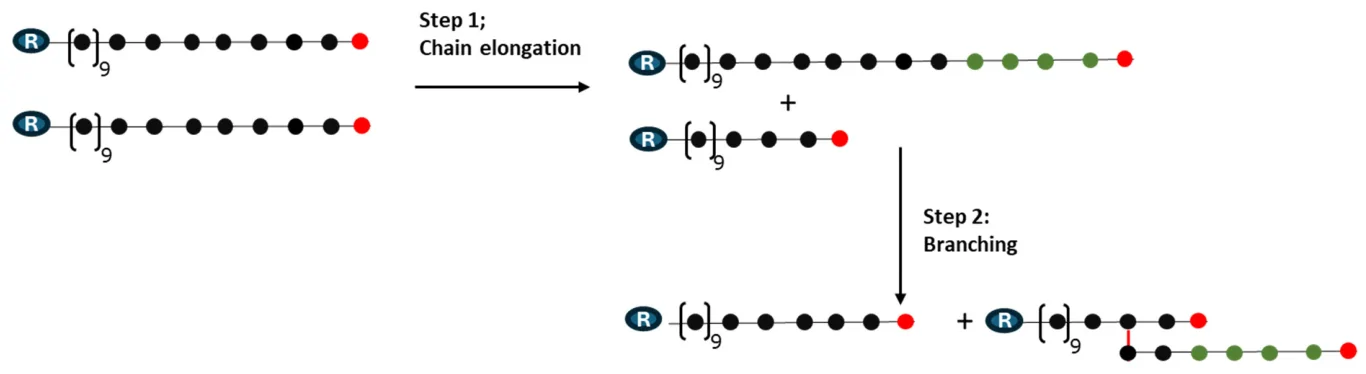
Two-step mechanism of chain elongation followed by branching catalysed by the *Butyrivibrio fibrisolvens* branching enzyme (BE). Blue dots represent reducing ends, while red dots indicate non-reducing ends. Green dots denote α-1,4-glucosidic moieties involved in chain elongation via transglucosylation. The red line represents the newly formed α-1,6-glucosidic bonds generated during the branching step. Black dots represent glycosidic moieties within the glucan backbone. The number 9 corresponds to the additional glucose residues required to extend the chain to DP18 (maltooctadecaose) [27].

Intriguingly, the GH13 BE from *Butyrivibrio fibrisolvens* appears to follow a sequential two-step catalytic process when acting on maltooctadecaose, in which an initial α-(1→4)-transglycosylation reaction elongates α-glucan chains [74]. This elongation activity is proposed to arise from an alternative acceptor-binding mode within the extended catalytic cleft, where the non-reducing end of the acceptor glucan is oriented toward the catalytic center, enabling α-(1→4)-glycosyl transfer. Thus, reaction specificity is governed by acceptor positioning and hydroxyl group accessibility within the active site [75]. The newly formed longer chains subsequently serve as acceptor substrates for the branching reaction (Figure 5). This mechanism links elongation and branching activities, with elongation generating suitable acceptor chains that facilitate subsequent branch formation. Notably, the branched glucan product generated by this BE represents one of the few reported cases in which BE treatment results in an increase in molecular weight (Mw).

These findings demonstrate that secondary catalytic activities are not merely statistical outcomes of a single mechanism, but integral components of the enzymatic restructuring process in which different activities enhance substrate availability for one another.

This behaviour of BEs is not unique among GH transferase-active enzymes, as recently demonstrated for other transferases, such as the recombinant maltotriosyltransferase (MTase) from *Aeribacillus pallidus* [55]. This enzyme introduces both α-1,3 and α-1,6 glucosidic linkages into corn starch, resulting in a “hybrid-branched” architecture that alters digestibility kinetics.

## 3. Dual Enzymatic Approaches

The cooperation between chain elongation and branching has also been demonstrated using 4α-GTs in combination with BEs, initially in cascade systems and more recently in simultaneous reactions [76,77]. Moreover, this dual-enzyme strategy is not limited to this specific enzyme pair but appears to be broadly applicable across different enzyme combinations [58,78,79,80].

Dual enzymatic approaches can be performed in cascade, where each enzyme modifies the starch structure to generate suitable substrates for subsequent enzymatic action. When enzymes are applied simultaneously, a key requirement is overlapping operational conditions, such as pH and temperature, allowing both enzymes to remain catalytically active.

### 3.1. Coupled Catalysis of BE and 4αGTs

Branching generally occurs only for donor chains above a defined degree of polymerization (DP), with a commonly accepted lower limit of DP > 8 [30,81]. For RoBE, a minimum of DP ≥ 10 has been reported for efficient branching. As branching proceeds, donor chains are shortened, reducing the number of substrates that meet this threshold and thereby limiting further branching. In this context, disproportionation and chain elongation catalysed by 4-α-glucanotransferase (4-αGT) can redistribute chain lengths within the glucan population, increasing the DP of specific chains and thereby restoring their suitability as substrates for branching enzyme activity.

A sequential three-step process employing the branching enzyme from RoBE and a 4αGT from *T*. *thermophilus* was shown to increase branching density [76,77]. In this approach, RoBE initially introduces branch points, thereby increasing substrate availability for 4αGT. Subsequently, 4αGT redistributes glucan chain lengths through disproportionation reactions, generating elongated chains that serve as new acceptor substrates for further branching during the final RoBE treatment step.

Furthermore, the co-application of 4αGT with BEs from *R*. *marinus* (GH13) and *T*. *thermophilus* (GH57) resulted in a synergistic increase in branching density, demonstrating cooperative activity between BEs and 4αGT [78]. More recently [58], similar effects were observed for the combination of RoBE and a 4αGT from *Thermus aquaticus*, suggesting that such synergism may represent a general feature of branching enzyme–4αGT systems operating under compatible reaction conditions.

**Figure 6:**
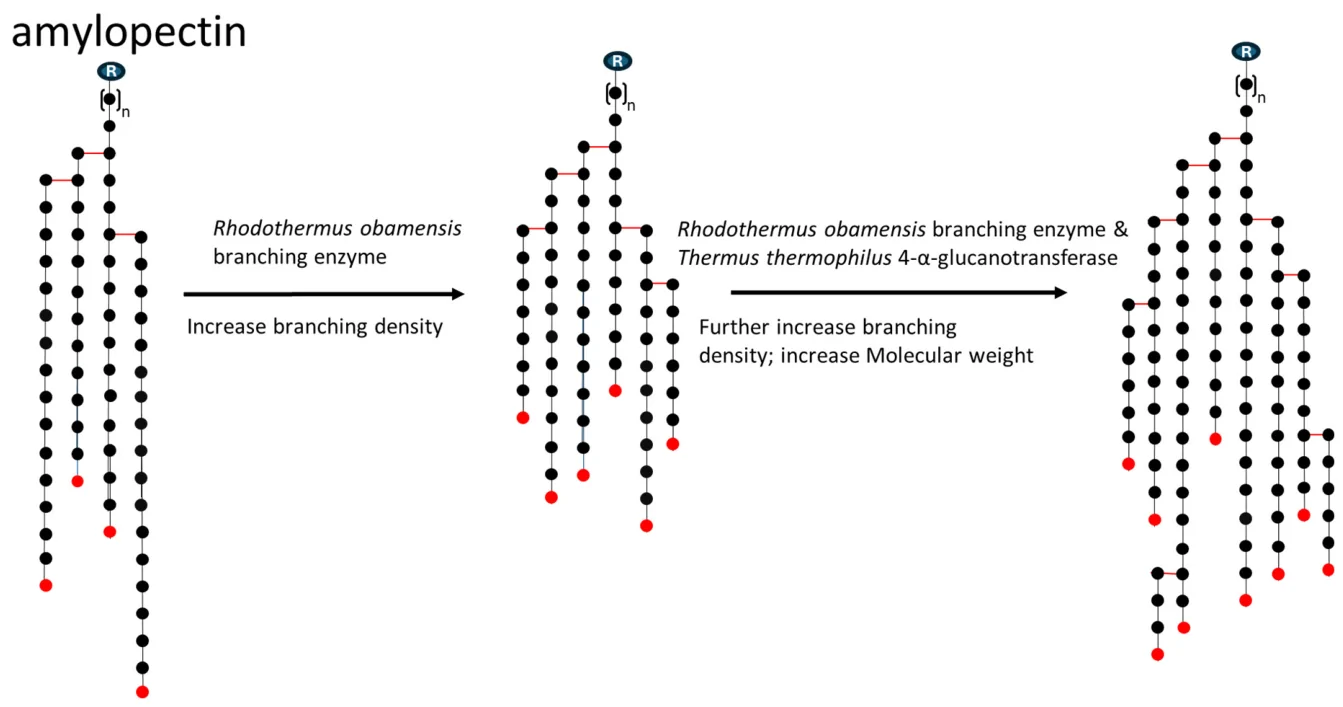
Comparison of native amylopectin with *Rhodothermus obamensis* branching enzyme (RoBE)-treated amylopectin and subsequent combined treatment with RoBE and *Thermus thermophilus* 4-α-glucanotransferase. Red dots represent non-reducing ends, blue dots represent reducing ends, and black dots indicate anhydrous glucose units within the glucan backbone. n denotes the degree of polymerisation (DP) of an α-glucan chain. Red lines denote α-(1→6) branch linkages [58].

In the latter study, the synergistic action of BE and 4αGT resulted in increases in both branching density and Mw (Figure 6). This was attributed to 4αGT consuming low-Mw oligomers and elongating chains to approximately DP10, consistent with its activity on substrates > DP3. Subsequent catalytic competition favours branching due to the irreversible nature of the branching reaction by BE over the reversible disproportionation by 4αGT. This results in an alternating cycle of elongation and branching. Overall, low-MW species are progressively consumed in a polymerization-like process.

This highlights the potential of collaborative enzyme ensembles. True polymerization has mainly been reported for sucrose using GH32 and GH70 enzymes from lactic acid bacteria, producing alternan, reuteran, mutan, dextran, and fructans such as levan [82,83,84], but not for GH13 systems (except for amylosucrase EC 2.4.1.4, short chain amylose synthesis from sucrose). The RoBE (GH13)/4αGT (GH77) ensemble demonstrates how enzyme cooperation can generate functions and distinct glucan structures not achievable by the individual enzymes. Compared to branching by RoBE alone, this dual enzymatic ensemble produces α-glucans with higher α-amylolytic resistance, consistent with increased branching density. Moreover, collaborative interactions among different enzymes have also been reported on different types of BEs [74,85].

### 3.2. BEs Collaborative Enzyme Ensembles

An illustrative example of interplaying BE activity is found in starch biosynthesis, where multiple isoforms act synergistically. In plants such as *Oryza sativa*, BEI transfers longer glucan chains, while BEII isoforms introduce short branches (e.g., DP6–7) [85]. This division of activity creates a cooperative system in which products generated by one enzyme are further processed by another. As a result, branching is highly structured, producing the defined chain-length distribution characteristic of amylopectin.

A cooperative BE system has also been demonstrated in vitro. The co-application of GH13 and GH57 BEs from *P*. *mobilis* to maltooctadecaose (MD18) resulted in a synergistic increase in branching density compared to either enzyme alone [38]. This effect is attributed to the hydrolytic side activity of the GH57 BE, which generates additional acceptor substrates. These newly generated chains enhance GH13 BE catalytic efficiency, showing a functional interplay where one enzyme supplies substrates and the other drives branching, which GH13 BE alone cannot achieve.

These examples illustrate how multiple enzymes can cooperatively generate new polymer architectures and therefore warrant further investigation, as nature similarly relies on coordinated multi-enzyme systems in glycogen and starch biosynthesis.

Many studies on branching and transferase active enzymes focus on their physiological effects, especially glucose release during small intestinal digestion. BE-modified glucans are often linked to a lower glycaemic index and a slower, more sustained glucose release.

## 4. α-Glucan Architecture and Digestibility

Most transferase-active starch-modifying enzymes are applied in food-related applications. As these modified starches are intended for human consumption, their digestibility, linked to glucosidic bond ratios and chain-length distribution, has been a primary focus in the discovery and study of BEs.

### 4.1. Effects of BE-Modified Starches on Digestion Kinetics

Starch is a natural energy source for the human body because it provides glucose upon digestion. Its breakdown is carried out by digestive enzymes, including salivary alpha-amylase, pancreatic alpha-amylase, and brush-border enzymes in the small intestine [86,87].

In vitro starch digestibility is commonly assessed using the Englyst method [88,89] and INFOGEST protocol, which quantify time-dependent glucose release. Based on hydrolysis kinetics, starch is classified as rapidly digestible (RDS, <20 min), slowly digestible (SDS, 20–120 min), or resistant starch (RS, >120 min), with RS escaping small intestinal digestion and reaching the colon for microbial fermentation. Pregelatinized native starch is predominantly RDS.

In contrast, BE-treated starches exhibit biphasic digestion kinetics as a result of a shift toward low-Mw glucans and enrichment of α-limit dextrins (DP > 8) containing α-1,6 linkages, which are more resistant to α-amylolytic hydrolysis [56,90,91]. These highly branched, high-Mw structures further slow down small-intestinal enzymatic degradation to glucose, thereby increasing slowly digestible starch (SDS). This profile is consistently observed across BEs from *T*. *kodakarensis*, *R*. *marinus*, *R obamensis*, and *P*. *mobilis* [91,92]. In addition, a minor fraction (<10%) of branched glucans remains resistant within 120 min.

Cooperative enzyme ensembles such as RoBE/4αGT enhance α-limit dextrin formation, further increasing slowly digestible starch (SDS) and resistant starch (RS) levels (≈10–20%) compared to single BE treatment [56,57,92]. These results indicate that parts of the glucan structure resist enzymatic digestion due to kinetic limitations or structural inaccessibility. However, the mechanisms driving the transition from SDS to RS remain unclear and require further investigations into branched glucan structural stability during enzymatic processing.

Although most studies on BE-modified starches are performed in vitro, the available in vivo investigations indicate comparable effects, including delayed glucose release, improved postprandial glucose control, and prolonged glucose availability [91,93,94].

Early-stage digestion processes also deserve greater attention, as BE-induced structural modifications may enhance glucan integrity and alter susceptibility to α-amylase during the initial phases of digestion [72]. The resulting retention of structure during mastication and oral processing could influence textural and sensory properties, potentially improving mouthfeel quality [72,95]. These effects remain largely unexplored and may represent an additional avenue for expanding the application potential of BE-modified carbohydrates in food systems.

Modulating these properties via controlled BE kinetics, coupled primary and secondary reactions, and enzyme ensembles, collectively termed “enzymatic glucan engineering”, warrants further investigation. Notably, BEs are not the only GH family enzymes capable of forming α-1,6 linkages.

### 4.2. Different Enzymatic Pathways for α-(1→6)-glycosidic Bond Formation and Their Influence on Digestibility

The BEs involved in starch and glycogen biosynthesis primarily belong to GH13 and GH57. Approximately a decade ago, a small group of GH70 enzymes exhibiting branching activity on starch and maltodextrins in vitro were identified [9]. Although GH70 enzymes typically catalyse the conversion of sucrose into α-glucan polymers [84], three GH70 subfamilies, designated GtfB, GtfC, and GtfD, utilize starch and maltodextrins as donor substrates and are unable to use sucrose [8,9,10].

GtfD enzymes, particularly 4,6-α-glucanotransferases, transfer α-(1→4)-linked glucan segments to the C6 hydroxyl of glucose residues, forming highly branched α-glucan structures with up to 18% α-(1→6) branches [8]. Unlike classical branching enzymes (GH13 and GH57), GtfD enzymes can also utilize non-reducing chain ends as acceptors, forming linear α-(1→6)-linked segments. Depending on the enzyme, up to ~18% of linear α-(1→6) linkages are formed. Thus, the glucan products consist of up to 36% α-(1→6) linkages.

In addition, GtfD enzymes can transfer short donor fragments (e.g., maltose), whereas GH13 and GH57 branching enzymes typically require longer donor chains. As a result, GtfD products display higher branching (~18% α-(1→4,6) linkages), altered chain-length distributions, and a bimodal molecular weight profile, comprising a low-Mw fraction (~10 kDa) and a high-Mw fraction (~10,000 kDa). These catalytic features yield α-glucans that are structurally distinct from those produced by GH13 and GH57 enzymes. As with classical BEs, increased α-(1→6) content reduces enzymatic digestibility by limiting α-amylase accessibility.

However, systematic comparative studies evaluating structural characteristics and digestion behaviour across GH13/GH57 BEs and GH70 4,6-α-glucanotransferases (GtfD) remain scarce. Such comparisons could provide critical insights into the role of α-1,6 content and glucan architecture in determining digestion kinetics and structure–function relationships.

Research on starch-modifying enzymes, including BEs, has predominantly focused on food applications, particularly in relation to physiological properties and functional performance. More broadly, starch-modifying enzymes have been studied mainly for food functionality, despite their physicochemical properties being potentially relevant for industrial applications.

## 5. BE-Modified Starches: Beyond Food Applications

### 5.1. BE-Modified Starches as Biobased Alternatives to Synthetic Water-Soluble Polymers

Beyond food applications, starch is widely used in industrial processes such as corrugating adhesives and paper processing [96], yet its broader potential remains somewhat underexploited. In high-solid systems, native starch upon storage is susceptible to recrystallisation, leading to poor solubility and viscosity instability [97]. This limits its use in storage-stable applications such as personal and home care. As a result, non-biodegradable petrochemical-derived polymers (e.g., polyacrylates, polyvinyl alcohol, and polyquaterniums) still dominate due to functional reliability despite sustainability concerns [98,99]. This underscores an opportunity for enzymatic glucan engineering to develop structurally tailored, storage-stable, biodegradable starch-based alternatives.

BE treatment increases branching density and modulates Mw wherein Mw reduction enhances solubility, while increased branching suppresses retrogradation by limiting chain reassociation over time [100]. This further improves storage stability and may also benefit systems subjected to cooling or freeze–thaw cycles, as increased branching reduces starch retrogradation and syneresis, thereby enhancing freeze–thaw stability [101]. These BE-induced characteristics show potential for partial replacement of synthetic binders in paper coating formulations, where at least 50% of conventional latex binders can be substituted with BE-treated starches while maintaining or even improving printability [102,103,104,105].

In replacing water-soluble polymers, a key challenge is achieving specific functionalities, as most synthetic polymers carry charge or exhibit a defined hydrophilic–hydrophobic balance, whereas starch is inherently hydrophilic and largely uncharged. A practical strategy to overcome this limitation is combined enzymatic–chemical derivatisation.

### 5.2. Enzymatic Catalysis Followed by Chemical Derivatization

Dual-modified polysaccharides can be produced via enzymatic restructuring followed by chemical derivatisation, combining biological precision with chemical versatility. This approach combines structural control from enzymatic remodelling with functional diversity from chemical modification [106,107,108,109]. However, these processes typically occur in solution, making downstream purification, including separation and removal of residual reagents, more cost-intensive. In addition, higher degrees of chemical modification may improve functionality but reduce biodegradability, limiting overall sustainability.

While remaining within biodegradability constraints, recent research [56,72] shows that the substrate scope of BE can be expanded beyond native starches, enabling the redistribution of chemically modified α-glucans.

### 5.3. Enzymatic Branching Subsequent to Chemical Modification of Starch

Recent studies [56,72] demonstrate that BEs can introduce branching into solubilised TEMPO-oxidized, acetylated, and hydroxypropylated starches, beyond native pregelatinised starch, reflecting higher substrate adaptability than expected. In contrast to subsequent chemical modification of BE-treated starches, this approach avoids costly separation steps, as residual reagents are already removed during the initial chemical suspension process, thereby reducing cost- and energy-intensive processing steps.

At higher DS, RoBE retains hydrolytic activity, although this decreases progressively above DS ~0.2. Branching occurs only when Mw is reduced below ~230 kDa, reflecting the coupling between hydrolysis and transglucosylation, as observed for native starch [72]. Rather than a strict limitation, this defines a design space in which biopolymers can be tuned to meet sustainability and biodegradability requirements, with chemical modification applied to a controlled extent.

**Figure 7:**
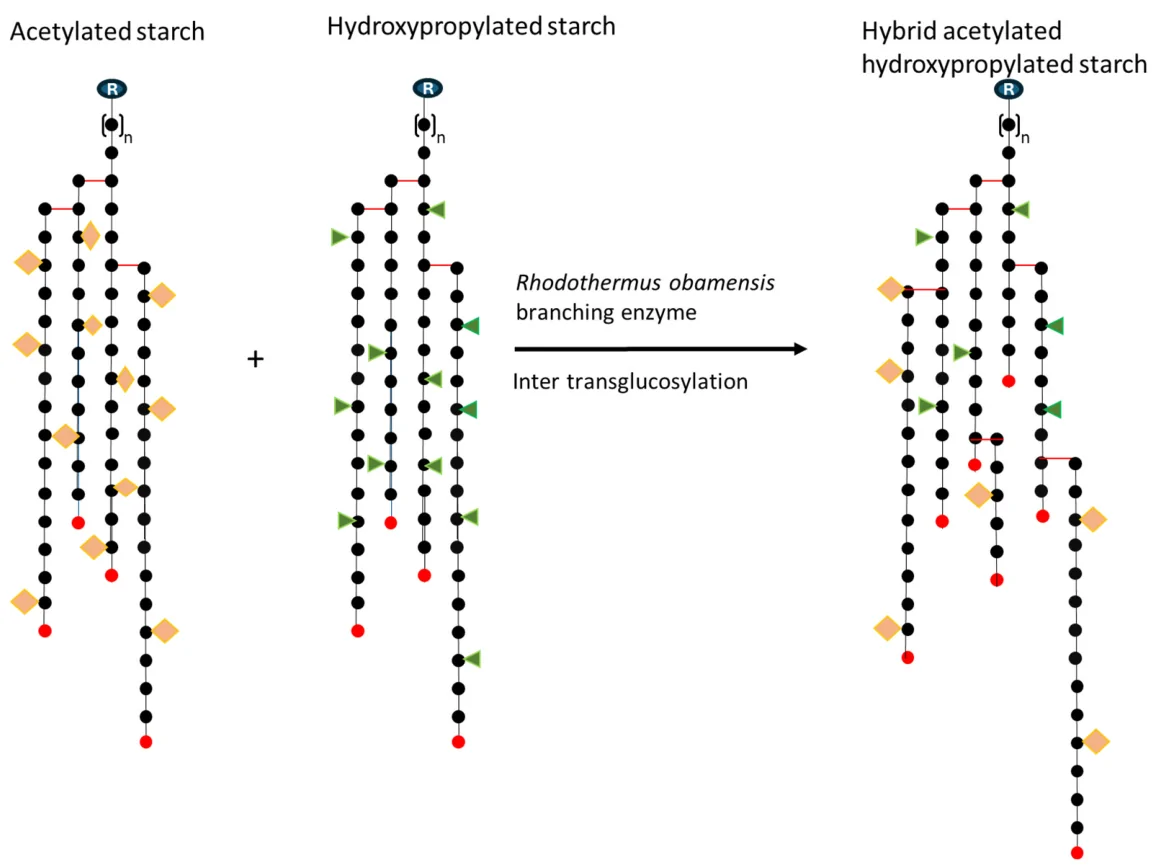
Starch hybrid structures formed through intermolecular transglucosylation catalysed by the *Rhodothermus obamensis* branching enzyme between hydroxypropylated and acetylated starch chains. Diamonds represent acetyl substituents, green triangles hydroxypropyl substituents, blue dots reducing ends, red dots non-reducing ends, black dots glucosyl units, and red lines α-1,6-glucosidic bonds.

Intriguingly, chemically modified starches enable a clearer distinction between intra- and inter-transglucosylation, which is difficult to observe in native starch due to its structural uniformity and is therefore likely underappreciated. When branching reactions are applied to modified starches, these processes become more apparent, particularly in blends of different chemically modified substrates. RoBE acts on such blends, leading to the formation of hybrid glucan structures and providing evidence for inter-transglucosylation [56], as illustrated in Figure 7. These hybrid structures are difficult to obtain via chemical modification.

These findings provide new routes for starch modification, in which structural motifs from different starch derivatives are combined into a single molecular framework via enzymatic branching.

RoBE acts on chemically modified starches [56], enabling diverse structural combinations. This approach combines the benefits of branching, including improved storage stability through reduced retrogradation and altered polymer–solvent interactions that enable higher solid concentrations due to reduced viscosity, with the functionality introduced by chemical substituents such as hydrophobicity and charge. This is particularly relevant for replacing synthetic polymers, as polymer composition, topology, and chemical functionality determine performance across a wide range of applications. Enzymatic branching enables the design of bio-based, biodegradable polymers with tailored solubility, charge, hydrophobicity, and molecular architecture, which are key properties for personal and home care applications.

### 5.4. Time-Dependent Behaviour of BE-Modified α-Glucans in Pharmaceutical Excipient Applications

BEs induces molecular-level changes in Mw, α-1,6 glycosidic bond ratio, and chain length distribution, which influence higher-order organization and thus hydrodynamic volume and solubility behaviour as described by Hansen solubility parameters (HSP). HSP describe intermolecular interactions governing material compatibility, miscibility, and solubility. They are particularly useful for predicting ingredient compatibility in aqueous formulations and guiding the replacement of synthetic polymers [110]. Some of these characteristics are time-dependent and may evolve under application conditions [72]. Structural integrity can therefore be classified as dynamic (time-dependent) or static (time-independent).

In dynamic systems such as the human digestive tract, branching modulates structural integrity during digestion, leading to slower glucose release [111]. Similar principles are used in pharmaceutical systems for controlled drug release, carrier, and bioactive encapsulation. A two-step enzymatic process involving BEs and 4αGT yields starches with longer and more abundant α-1,6 branches than native or single-enzyme systems [57]. Combined with alginate, these materials form matrices for controlled and targeted delivery, highlighting the broader application potential of enzymatically modified starches. While a sequential application of BE and α-4GT was selected in this study, the reported benefits of their simultaneous application [58] suggest that products generated through such combined processes may exhibit comparable performance in these types of applications.

Another example in pharmaceutical applications is the subsequent modification of BE-treated starch with octenyl succinic anhydride (OSA) [109], which has been shown to improve the emulsification and aqueous solubility of puerarin, a poorly water-soluble natural isoflavone. These findings further highlight the potential of BE-treated starches as functional carriers for drug delivery and encapsulation.

## 6. Conclusions

The catalytic mechanism of BEs exhibits far greater complexity than is captured by the main reaction outcome alone. Growing evidence demonstrates that BE catalysis is best described as networked catalysis, where concurrent and sequential reactions collectively define the final α-glucan architecture. A mechanistic understanding of this network enables control over substrate specificity and mechanism-guided tuning of glucan structure across scales.

This enzymatic glucan engineering framework provides a toolbox for constructing tailored architectures for targeted applications, extending functionality into domains currently inaccessible to conventional starch derivatives. Water-borne formulations in personal and home care products, long dominated by synthetic polymers and stringent stability requirements, may be reformulated using branching of chemically modified starches that stabilise solution behaviour over time.

Another avenue for expanding the function of BEs lies in their collaborative interactions with other GH enzymes, where branching and complementary activities such as disproportionation can lead to polymerisation-like effects. In contrast to the activity of individual enzymes, these synergistic systems can induce structural outcomes reminiscent of conventional polymerisation processes. This highlights the broader potential of biocatalysis within GH enzyme networks, particularly for establishing novel glucan structures.

Within the food domain, the transition from slowly digestible to resistant starch represents a key target for transferases that form different glycosidic bonds, including BEs. Accordingly, enzymatic glucan engineering extends beyond the control of glucose release, enabling modulation of functional properties such as texture, thereby establishing physicochemical–functional relationships that are currently unattainable with existing resistant starches.

Taken together, these developments reposition enzymatic starch modification not as a niche processing technology, but as a foundational platform for the design of sustainable polymeric materials with tuneable structure–function relationships spanning both food and non-food applications.

## Figures and Tables

**Table 1:** Overview of branching enzymes from GH13 and GH57, including GH70 GtfD enzyme.

Enzyme Family	Enzyme Name	Organism & Literature Reference	Catalytic Fold	EC Number
GH13	GlgB	*Escherichia coli* [30]	(β/α)_8_ barrel	EC 2.4.1.18
GH13	GlgB	*Salmonella enterica* [31]	(β/α)_8_ barrel	EC 2.4.1.18
GH13	GlgB	*Bacillus subtilis* [32]	(β/α)_8_ barrel	EC 2.4.1.18
GH13	GlgB	*Bacillus stearothermophilus* [33]	(β/α)_8_ barrel	EC 2.4.1.18
GH13	GlgB	*Bacillus cereus* [33]	(β/α)_8_ barrel	EC 2.4.1.18
GH13	GBEDr	*Deinococcus radiodurans* [34]	(β/α)_8_ barrel	EC 2.4.1.18
GH13	BfGBE	*Butyrivibrio fibrisolvens* [35]	(β/α)_8_ barrel	EC 2.4.1.18
GH13	GBE Dg	*Deinococcus geothermalis* [34]	(β/α)_8_ barrel	EC 2.4.1.18
GH13	RmGBE	*Rhodothermus marinus* [36]	(β/α)_8_ barrel	EC 2.4.1.18
GH13	RoBE	*Rhodothermus obamensis* [37]	(β/α)_8_ barrel	EC 2.4.1.18
GH13	PmGBE13	*Petrotoga mobilis* [38]	(β/α)_8_ barrel	EC 2.4.1.18
GH13	CgGlgB	*Corynebacterium glutamicum* [39]	(β/α)_8_ barrel	EC 2.4.1.18
GH13	ScoGlgB	*Streptomyces coelicolor* [40]	(β/α)_8_ barrel	EC 2.4.1.18
GH13	PaGlgB	*Pseudomonas aeruginosa* [41]	(β/α)_8_ barrel	EC 2.4.1.18
GH13	MtGlgB	*Mycobacterium tuberculosis* [42]	(β/α)_8_ barrel	EC 2.4.1.18
GH13	VcGlgB	*Vibrio cholerae* [43]	(β/α)_8_ barrel	EC 2.4.1.18
GH13	EcGlgB	*Enterobacter cloacae* [44]	(β/α)_8_ barrel	EC 2.4.1.18
GH13	SBEI	*Zea mays* [45]	(β/α)_8_ barrel	EC 2.4.1.18
GH13	SBEIIa	*Zea mays* [46]	(β/α)_8_ barrel	EC 2.4.1.18
GH13	SBEIIb	*Zea mays* [47]	(β/α)_8_ barrel	EC 2.4.1.18
GH13	AtSBE2	*Arabidopsis thaliana* [48]	(β/α)_8_ barrel	EC 2.4.1.18
GH57	TkGBE57	*Thermococcus kodakarensis* [49]	(β/α)_7_ barrel	EC 2.4.1.18
GH57	PmGBE57	*Petrotoga mobilis* [38]	(β/α)_7_ barrel	EC 2.4.1.18
GH57	MtGlgB57	*Mycobacterium tuberculosis* [50]	(β/α)_7_ barrel	EC 2.4.1.18
GH57	MgGBE57	*Meiothermus* sp. [27]	(β/α)_7_ barrel	EC 2.4.1.18
GH57	SsGBE57	*Sulfolobus solfataricus* [51]	(β/α)_7_ barrel	EC 2.4.1.18
GH57	PfGBE57	*Pyrococcus furiosus* [52]	(β/α)_7_ barrel	EC 2.4.1.18
GH57	TeGBE57	*Thermotogae* sp. [53]	(β/α)_7_ barrel	EC 2.4.1.18
GH57	TtGBE57	*Thermus thermophilus* [50]	(β/α)_7_ barrel	EC 2.4.1.18
GH57	TmGBE57	*Thermotoga maritima* [53]	(β/α)_7_ barrel	EC 2.4.1.18
GH70	GtfD	*Paenibacillus beijingensis* [8]	circularly permuted (β/α)_8_	EC 2.4.1.394

## Data Availability

No new data were created or analyzed in this study. Data sharing is not applicable to this article. The authors utilized AI-assisted technology exclusively to enhance the manuscript’s readability and language, while thoroughly examining and editing the output themselves. They retain full responsibility for the content and accuracy of the entire article.

## References

[1] Leemhuis H., Kelly R.M., Dijkhuizen L. (2010). Engineering of cyclodextrin glucanotransferases and the impact for biotechnological applications. Appl. Microbiol. Biotechnol..

[2] van der Maarel M.J.E.C., van der Veen B., Uitdehaag J.C.M., Leemhuis H., Dijkhuizen L. (2002). Properties and applications of starch-converting enzymes of the alpha-amylase family. J. Biotechnol..

[3] Van der Maarel M.J.E.C., Leemhuis H. (2013). Starch modification with microbial alpha-glucanotransferase enzymes. Carbohydr. Polym..

[4] Van der Maarel M.J.E.C., Vos A., Sanders P., Dijkhuizen L. (2003). Properties of the glucan branching enzyme of the hyperthermophilic bacterium aquifex aeolicus. Biocatal. Biotransform..

[5] Christensen S.J., Madsen M.S., Zinck S.S., Hedberg C., Sorensen O.B., Svensson B., Meyer A.S. (2023). Enzymatic potato starch modification and structure-function analysis of six diverse GH77 4-alpha-glucanotransferases. Int. J. Biol. Macromol..

[6] Henrissat B. (1991). A classification of glycosyl hydrolases based on amino acid sequence similarities. Biochem. J..

[7] Drula E., Garron M.L., Dogan S., Lombard V., Henrissat B., Terrapon N. (2022). The carbohydrate-active enzyme database: Functions and literature. Nucleic Acids Res..

[8] Gangoiti J., Lamothe L., van Leeuwen S.S., Vafiadi C., Dijkhuizen L. (2017). Characterization of the *Paenibacillus beijingensis* DSM 24997 GtfD and its glucan polymer products representing a new glycoside hydrolase 70 subfamily of 4,6-alpha-glucanotransferase enzymes. PLoS ONE.

[9] Gangoiti J., Pijning T., Dijkhuizen L. (2016). The Exiguobacterium sibiricum 255-15 GtfC Enzyme Represents a Novel Glycoside Hydrolase 70 Subfamily of 4,6-alpha-Glucanotransferase Enzymes. Appl. Environ. Microbiol..

[10] Gangoiti J., van Leeuwen S.S., Gerwig G.J., Duboux S., Vafiadi C., Pijning T., Dijkhuizen L. (2017). 4,3-alpha-Glucanotransferase, a novel reaction specificity in glycoside hydrolase family 70 and clan GH-H. Sci. Rep..

[11] Dong J., Abou Hachem M., Wang Y., Li X., Zhang B., Pijning T., Svensson B., Dijkhuizen L., Jin Z., Bai Y. (2024). Tailor-Made alpha-Glucans by Engineering the Processivity of alpha-Glucanotransferases via Tunnel-Cleft Active Center Interconversions. J. Agric. Food Chem..

[12] Dong J., Bai Y., Wang Q., Chen Q., Li X., Wang Y., Ji H., Meng X., Pijning T., Svensson B. (2024). Insights into the Structure-Function Relationship of GH70 GtfB alpha-Glucanotransferases from the Crystal Structure and Molecular Dynamic Simulation of a Newly Characterized Limosilactobacillus reuteri N1 GtfB Enzyme. J. Agric. Food Chem..

[13] Bai Y., Gangoiti J., Dijkstra B.W., Dijkhuizen L., Pijning T. (2017). Crystal Structure of 4,6-alpha-Glucanotransferase Supports Diet-Driven Evolution of GH70 Enzymes from alpha-Amylases in Oral Bacteria. Structure.

[14] Artola M., Wouters S., Schroder S.P., de Boer C., Chen Y., Petracca R., van den Nieuwendijk A., Aerts J., van der Marel G.A., Codee J.D.C. (2019). Direct Stereoselective Aziridination of Cyclohexenols with 3-Amino-2-(trifluoromethyl)quinazolin-4(3H)-one in the Synthesis of Cyclitol Aziridine Glycosidase Inhibitors. Eur. J. Org. Chem..

[15] Speciale G., Thompson A.J., Davies G.J., Williams S.J. (2014). Dissecting conformational contributions to glycosidase catalysis and inhibition. Curr. Opin. Struct. Biol..

[16] Zong Z., Zhang X., Chen P., Fu Z., Zeng Y., Wang Q., Chipot C., Leggio L.L., Sun Y. (2024). Elucidation of the noncovalent interactions driving enzyme activity guides branching enzyme engineering for alpha-glucan modification. Nat. Commun..

[17] Gaenssle A. (2020). The Versatile Activity of Glycogen Branching Enzymes. Ph.D. Thesis.

[18] Bissaro B., Durand J., Biarnés X., Planas A., Monsan P., O’Donohue M.J., Fauré R. (2015). Molecular Design of Non-Leloir Furanose-Transferring Enzymes from an α-l-Arabinofuranosidase: A Rationale for the Engineering of Evolved Transglycosylases. ACS Catal..

[19] Desmet T., Soetaert W., Bojarova P., Kren V., Dijkhuizen L., Eastwick-Field V., Schiller A. (2012). Enzymatic glycosylation of small molecules: Challenging substrates require tailored catalysts. Chemistry.

[20] Yang C., Pijning T., Jurak E. (2026). Identification of Key Residues Governing Branch Activity and Acceptor Substrate Binding in GH13_8 Glycogen Branching Enzymes. J. Agric. Food Chem..

[21] Yang C., Poláček A., Pijning T., van der Maarel M.J.E.C., Jurak E. (2026). Key Loops in GH13_8 Subfamily Glycogen Branching Enzymes Modulate Their Branch Length Preference. ACS Catal..

[22] Davies G.J., Gloster T.M., Henrissat B. (2005). Recent structural insights into the expanding world of carbohydrate-active enzymes. Curr. Opin. Struct. Biol..

[23] Janecek S., Svensson B., MacGregor E.A. (2014). Alpha-Amylase: An enzyme specificity found in various families of glycoside hydrolases. Cell. Mol. Life Sci..

[24] Stam M.R., Danchin E.G., Rancurel C., Coutinho P.M., Henrissat B. (2006). Dividing the large glycoside hydrolase family 13 into subfamilies: Towards improved functional annotations of alpha-amylase-related proteins. Protein Eng. Des. Sel..

[25] Zhang X., Leemhuis H., van der Maarel M. (2019). Characterization of the GH13 and GH57 glycogen branching enzymes from *Petrotoga mobilis* sj95 and potential role in glycogen biosynthesis. PLoS ONE.

[26] Zhang X., Leemhuis H., van der Maarel M. (2019). Synthesis of highly branched alpha-glucans with different structures using gh13 and gh57 glycogen branching enzymes. Carbohydr. Polym..

[27] Bax H.H.M., van der Maarel M., Jurak E. (2023). Alpha-1,4-transglycosylation Activity of GH57 Glycogen Branching Enzymes Is Higher in the Absence of a Flexible Loop with a Conserved Tyrosine Residue. Polymers.

[28] Xiao Y., Kong H., Zhang Z., Li C., Ban X., Gu Z., Li Z. (2024). A recombinant amylomaltase from *Thermus thermophilus* STB20 can tailor the macromolecular characteristics of tapioca starch through its transglycosylation activity. Food Biosci..

[29] Kaewpathomsri P., Takahashi Y., Nakamura S., Kaulpiboon J., Kidokoro S.-I., Murakami S., Krusong K., Pongsawasdi P. (2015). Characterization of amylomaltase from Thermus filiformis and the increase in alkaline and thermo-stability by E27R substitution. Process Biochem..

[30] Ahmad N., Mehboob S., Rashid N. (2015). Starch-processing enzymes—Emphasis on thermostable 4-α-glucanotransferases. Biologia.

[31] Baecker P.A., Greenberg E., Preiss J. (1986). Biosynthesis of bacterial glycogen. Primary structure of *Escherichia coli* 1,4-alpha-D-glucan:1,4-alpha-D-glucan 6-alpha-D-(1, 4-alpha-D-glucano)-transferase as deduced from the nucleotide sequence of the glg B gene. J. Biol. Chem..

[32] Kiel J.A.K.W., Boels J.M., Beldman G. (1991). Molecular cloning and nucleotide sequence of the glycogen branching enzyme gene (glgB) form *Bacillus stearothermophilus* and expression in *Escheria cob* and *Bacillus subtilis*. Mol. Gen. Genet..

[33] Takata H., Takaha T., Kuriki T., Okada S., Takagi M., Imanaka T. (1994). Properties and Active Center of the Thermostable Branching enzymes form *Bacillus stearothermophilus*. Appl. Environ. Microbiol..

[34] Palomo M., Kralj S., van der Maarel M.J., Dijkhuizen L. (2009). The unique branching patterns of deinococcus glycogen branching enzymes are determined by their N-terminal domains. Appl. Environ. Microbiol..

[35] Rumback E., Rawlings D., Lindsey G.G., Woods D.R. (1991). Characterisation of the *Butyrivibrio fibrisolvens* glgB Gene, Which Encodes a Glycogen Branching Enzyme with Starch-Clearing Activity. J. Bacteriol..

[36] Yoon S.A., Lee S.B., Moon T.W. (2008). Purification and characterization of branching specificity of a novel extracellular amylolytic enzyme from marine hyperthermophilic *Rhodothermus marinus*. J. Microbiol. Biotechnol..

[37] Sako Y., Takai Y.I.K., Uchida A.A., Katayama Y. (1996). *Rhodothemus obamensis* sp. Nov., a modern lineage of extremely thermophilic marine bacteria. Int. J. Syst. Bacteriol..

[38] Bax H.H.M., Gaenssle A.L., van der Maarel M., Jurak E. (2023). The synergistic effect of gh13 and gh57 gbes of *Petrotoga mobilis* results in alpha-glucan molecules with a higher branch density. Polymers.

[39] Seibold G.M., Breitinger K.J., Kempkes R., Both L., Kramer M., Dempf S., Eikmanns B.J. (2011). The glgB-encoded glycogen branching enzyme is essential for glycogen accumulation in *Corynebacterium glutamicum*. Microbiology.

[40] Bruton C.J., Plaskitt K.A., Chater K.F. (1995). Tissue-specific glycogen branching isoenzymes in a multicellular prokaryote, *Streptomyces coelicolor* A3(2). Mol. Microbiol..

[41] Woodcock S.D., Syson K., Little R.H., Ward D., Sifouna D., Brown J.K.M., Bornemann S., Malone J.G. (2021). Trehalose and alpha-glucan mediate distinct abiotic stress responses in *Pseudomonas aeruginosa*. PLoS Genet..

[42] Pal K., Kumar S., Sharma S., Garg S.K., Alam M.S., Xu H.E., Agrawal P., Swaminathan K. (2010). Crystal structure of full-length *Mycobacterium tuberculosis* H37Rv glycogen branching enzyme: Insights of N-terminal beta-sandwich in substrate specificity and enzymatic activity. J. Biol. Chem..

[43] Bourassa L., Camilli A. (2009). Glycogen contributes to the environmental persistence and transmission of *Vibrio cholerae*. Mol. Microbiol..

[44] Kumar A., Larsen C.E., Preiss J. (1986). Biosynthesis of bacterial glycogen. Primary structure of *Escherichia coli* ADP-glucose:Alpha-1,4-glucan, 4-glucosyltransferase as deduced from the nucleotide sequence of the glgA gene. J. Biol. Chem..

[45] Yao Y., Guiltinan M.J. (2004). Maize Starch-Branching Enzyme Isoforms and Amylopectin Structure. In the Absence of Starch-Branching Enzyme IIb, the Further Absence of Starch-Branching Enzyme Ia Leads to Increased Branching. Plant Physiol..

[46] Gao M., Kim K.N., Shannon J.C., Guiltinan M.J. (1997). Independent Genetic Control of Maize Starch-Branching Enzymes IIa and IIb (Isolation and Characterization of a Sbe2a cDNA). Plant Physiol..

[47] Yandeau-Nelson M.D., Laurens L., Shi Z., Xia H., Smith A.M., Guiltinan M.J. (2011). Starch-branching enzyme IIa is required for proper diurnal cycling of starch in leaves of maize. Plant Physiol..

[48] Morvan C., Halpern D., Kénanian G., Pathania A., Anba-Mondoloni J., Lamberet G., Gruss A., Gloux K. (2017). The Staphylococcus aureus FASII bypass escape route from FASII inhibitors. Biochimie.

[49] Murakami T., Kanai T., Takata H., Kuriki T., Imanaka T. (2006). A novel branching enzyme of the gh-57 family in the hyperthermophilic archaeon *Thermococcus kodakaraensis* kod1. J. Bacteriol..

[50] Palomo M., Pijning T., Booiman T., Dobruchowska J.M., van der Vlist J., Kralj S., Planas A., Loos K., Kamerling J.P., Dijkstra B.W. (2011). *Thermus thermophilus* glycoside hydrolase family 57 branching enzyme: Crystal structure, mechanism of action, and products formed. J. Biol. Chem..

[51] Santos C.R., Tonoli C.C., Trindade D.M., Betzel C., Takata H., Kuriki T., Kanai T., Imanaka T., Arni R.K., Murakami M.T. (2011). Structural basis for branching-enzyme activity of glycoside hydrolase family 57: Structure and stability studies of a novel branching enzyme from the hyperthermophilic archaeon *Thermococcus kodakaraensis* KOD1. Proteins.

[52] Comfort D.A., Chou C.J., Conners S.B., VanFossen A.L., Kelly R.M. (2008). Functional-genomics-based identification and characterization of open reading frames encoding alpha-glucoside-processing enzymes in the hyperthermophilic archaeon *Pyrococcus furiosus*. Appl. Environ. Microbiol..

[53] Zhang X., Leemhuis H., Janecek S., Martinovicova M., Pijning T., van der Maarel M. (2019). Identification of *Thermotoga maritima* MSB8 GH57 alpha-amylase AmyC as a glycogen-branching enzyme with high hydrolytic activity. Appl. Microbiol. Biotechnol..

[54] Tetlow I.J., Emes M.J. (2014). A review of starch-branching enzymes and their role in amylopectin biosynthesis. IUBMB Life.

[55] Yang T., Huang Y., Wang C., Luo S., Xu Y., Huang J., Jin M. (2026). Engineering dual alpha-1,3/alpha-1,6 glycosidic bonds in starch via a novel maltotriosyl transferase for enhanced slow digestion and stability. Carbohydr. Polym..

[56] Essers M.K.H., van den Broek L.A.M., Leemhuis H., Bitter J.H. (2025). On the performance of branching enzymes on chemically modified starches. Carbohydr. Polym..

[57] Chen Y., Wang Y., Tian Y., Svensson B., Blennow A. (2024). Enzymatic synthesis of long-branched or short-branched starches with uniform molecular size. Food Biosci..

[58] Essers M.K.H., Leemhuis H., van den Broek L.A.M., Ozobodo K., van Walsem M., Bitter J.H. (2026). Dual-enzyme-induced polymerization for the synthesis of hyperbranched, high-molecular-weight alpha-glucans. Carbohydr. Polym..

[59] Lee W.M., Song Y.B., Kim G.R., Park J.H., Kim H.S., Lee B.H. (2026). Clean-label approaches to produce slowly digestible rice flour via starch modification: Elucidating digestion mechanisms via small intestinal alpha-glucosidase and in vivo levels. Int. J. Biol. Macromol..

[60] Ban X., Dhoble A.S., Li C., Gu Z., Hong Y., Cheng L., Holler T.P., Kaustubh B., Li Z. (2020). Bacterial 1,4-α-glucan branching enzymes: Characteristics, preparation and commercial applications. Crit. Rev. Biotechnol..

[61] Punia Bangar S., Ashogbon A.O., Singh A., Chaudhary V., Whiteside W.S. (2022). Enzymatic modification of starch: A green approach for starch applications. Carbohydr. Polym..

[62] Takata H., Takaha T., Okada S., Hizukuri S., Takagi M., Imanaka T. (1996). Structure of the cyclic glucan produced from amylopectin by *Bacillus stearothermophilus* branching enzyme. Carbohydr. Res..

[63] Takata H., Takaha T., Okada S., Takagi M., Imanaka T. (1996). Cyclization reaction catalyzed by branching enzyme. J. Bacteriol..

[64] Takata H., Kato T., Takagi M., Imanaka T. (2005). Cyclization reaction catalyzed by *Bacillus cereus* branching enzyme, and the structure of cyclic glucan produced by the enzyme from amylose. J. Appl. Glycosci..

[65] Takata H., Takaha T., Nakamura H., Fujii K., Okada S., Takagi M. (1997). Production and some properties of a dextrin with a narrow size distribution by the cyclization reaction of branching enzyme. J. Ferment. Bioeng..

[66] Takata H., Ohdan K., Takaha T., Kuriki T., Okada S. (2003). Properties of branching enzyme from hyperthermophilic bacterium, aquifex aeolicus, and its potential for production of highly-branched cyclic dextrin. J. Appl. Glycosci..

[67] Li X., Wang Y., Wu J., Jin Z., Dijkhuizen L., Svensson B., Bai Y. (2024). Designing starch derivatives with desired structures and functional properties via rearrangements of glycosidic linkages by starch-active transglycosylases. Crit. Rev. Food Sci. Nutr..

[68] Ballschmiter M., Futterer O., Liebl W. (2006). Identification and characterization of a novel intracellular alkaline alpha-amylase from the hyperthermophilic bacterium *Thermotoga maritima* MSB8. Appl. Environ. Microbiol..

[69] Blesak K., Janecek S. (2012). Sequence fingerprints of enzyme specificities from the glycoside hydrolase family gh57. Extremophiles.

[70] Janecek S., Martinovicova M. (2020). New groups of protein homologues in the alpha-amylase family GH57 closely related to alpha-glucan branching enzymes and 4-alpha-glucanotransferases. Genetica.

[71] Polacek A., Lombard V., Coutinho P.M., Terrapon N., Janecek S. (2025). Dividing the alpha-amylase family GH57 of starch hydrolases and related enzymes into subfamilies using evolutionary, clustering and functional criteria. Int. J. Biol. Macromol..

[72] Essers M.K.H., van den Broek L.A.M., Boeriu C.G., Leemhuis H., Orumaniye C., Bitter J.H. (2026). Modulation of starch structure at molecular and supramolecular levels via interlinked hydrolytic and branching activities of *Rhodothermus obamensis* branching enzyme. Int. J. Biol. Macromol..

[73] Li Y., Li C.M., Gu Z.B., Cheng L., Hong Y., Li Z.F. (2019). Digestion properties of corn starch modified by α-d-glucan branching enzyme and cyclodextrin glycosyltransferase. Food Hydrocolloid.

[74] Gaenssle A.L.O., Bax H.H.M., van der Maarel M., Jurak E. (2021). GH13 glycogen branching enzymes can adapt the substrate chain length towards their preferences via alpha-1,4-transglycosylation. Enzym. Microb. Technol..

[75] Hayashi M., Suzuki R., Colleoni C., Ball S.G., Fujita N., Suzuki E. (2017). Bound Substrate in the Structure of Cyanobacterial Branching Enzyme Supports a New Mechanistic Model. J. Biol. Chem..

[76] Sorndech W., Meier S., Jansson A.M., Sagnelli D., Hindsgaul O., Tongta S., Blennow A. (2015). Synergistic amylomaltase and branching enzyme catalysis to suppress cassava starch digestibility. Carbohydr. Polym..

[77] Sorndech W., Sagnelli D., Meier S., Jansson A.M., Lee B.H., Hamaker B.R., Rolland-Sabate A., Hebelstrup K.H., Tongta S., Blennow A. (2016). Structure of branching enzyme- and amylomaltase modified starch produced from well-defined amylose to amylopectin substrates. Carbohydr. Polym..

[78] Xiang G. (2022). Dual Biological Role of Glycoside Hydrolase Family Fifty-Seven Glycogen Branching Enzymes. Ph.D. Thesis.

[79] Kajiura H., Kakutani R., Akiyama T., Takata H., Kuriki T. (2008). A novel enzymatic process for glycogen production. Biocatal. Biotransform..

[80] Kajiura H., Takata H., Akiyama T., Kakutani R., Furuyashiki T., Kojima I., Harui T., Kuriki T. (2011). In vitro synthesis of glycogen: The structure, properties, and physiological function of enzymatically-synthesized glycogen. Biologia.

[81] Roussel X., Lancelon-Pin C., Vikso-Nielsen A., Rolland-Sabate A., Grimaud F., Potocki-Veronese G., Buleon A., Putaux J.L., D’Hulst C. (2013). Characterization of substrate and product specificity of the purified recombinant glycogen branching enzyme of *Rhodothermus obamensis*. Biochim. Biophys. Acta.

[82] Molina M., Cioci G., Moulis C., Severac E., Remaud-Simeon M. (2021). Bacterial alpha-Glucan and Branching Sucrases from GH70 Family: Discovery, Structure-Function Relationship Studies and Engineering. Microorganisms.

[83] Chen Z., Ni D., Zhang W., Stressler T., Mu W. (2021). Lactic acid bacteria-derived alpha-glucans: From enzymatic synthesis to miscellaneous applications. Biotechnol. Adv..

[84] Li X., Wang X., Meng X., Dijkhuizen L., Liu W. (2020). Structures, physico-chemical properties, production and (potential) applications of sucrose-derived alpha-d-glucans synthesized by glucansucrases. Carbohydr. Polym..

[85] Vu N.T., Shimada H., Kakuta Y., Nakashima T., Ida H., Omori T., Nishi A., Satoh H., Kimura M. (2008). Biochemical and crystallographic characterization of the starch branching enzyme I (BEI) from *Oryza sativa* L.. Biosci. Biotechnol. Biochem..

[86] Martinez M.M., Li C., Okoniewska M., Mukherjee I., Vellucci D., Hamaker B. (2018). Slowly digestible starch in fully gelatinized material is structurally driven by molecular size and a and b1 chain lengths. Carbohydr. Polym..

[87] Ryu J.J., Li X., Lee E.S., Li D., Lee B.H. (2022). Slowly digestible property of highly branched alpha-limit dextrins produced by 4,6-alpha-glucanotransferase from Streptococcus thermophilus evaluated in vitro and in vivo. Carbohydr. Polym..

[88] Englyst H.N., Cummings J.H. (1984). Simplified method for the measurement of total non-starch polysaccharides by gas—Liquid-chromatography of constituent sugars as alditol acetates. Analyst.

[89] Morais R.A., Sousa H.M.S., Martins G.A.S. (2025). Application of the INFOGEST Protocol to Evaluate the Effects of Simulated In Vitro Gastrointestinal Digestion on Polyphenols and Antioxidant Potential of Four Native Amazonian Fruits. ACS Omega.

[90] Kittisuban P., Lee B.H., Suphantharika M., Hamaker B.R. (2014). Slow glucose release property of enzyme-synthesized highly branched maltodextrins differs among starch sources. Carbohydr. Polym..

[91] Lee B.H., Yan L., Phillips R.J., Reuhs B.L., Jones K., Rose D.R., Nichols B.L., Quezada-Calvillo R., Yoo S.H., Hamaker B.R. (2013). Enzyme-synthesized highly branched maltodextrins have slow glucose generation at the mucosal alpha-glucosidase level and are slowly digestible in vivo. PLoS ONE.

[92] Zhang X., Leemhuis H., van der Maarel M. (2020). Digestion kinetics of low, intermediate and highly branched maltodextrins produced from gelatinized starches with various microbial glycogen branching enzymes. Carbohydr. Polym..

[93] Shiraki T., Kometani T., Yoshitani K., Takata H., Nomura T. (2015). Evaluation of Exercise Performance with the Intake of Highly Branched Cyclic Dextrin in Athletes. Food Sci. Technol. Res..

[94] Furuyashiki T., Tanimoto H., Yokoyama Y., Kitaura Y., Kuriki T., Shimomura Y. (2014). Effects of ingesting highly branched cyclic dextrin during endurance exercise on rating of perceived exertion and blood components associated with energy metabolism. Biosci. Biotechnol. Biochem..

[95] Hadnađev M., Dapčević Hadnađev T., Dokić L., Pajin B., Torbica A., Šarić L., Ikonić P. (2014). Physical and sensory aspects of maltodextrin gel addition used as fat replacers in confectionery filling systems. LWT-Food Sci. Technol..

[96] Chemelli A., Gomernik F., Thaler F., Huber A., Hirn U., Bauer W., Spirk S. (2020). Cationic starches in paper-based applications—A review on analytical methods. Carbohydr. Polym..

[97] Yu S., Xu J., Zhang Y., Kopparapu N.K. (2014). Relationship between intrinsic viscosity, thermal, and retrogradation properties of amylose and amylopectin. Czech. J. Food Sci..

[98] The European Parliament and the Council of the European Union (2025). Regulation (EU) 2025/2365 of the European Parliament and of the Council of 12 November 2025 on Preventing Plastic Pellet Losses to Reduce Microplastic Pollution.

[99] Rozman U., Kalcikova G. (2021). The first comprehensive study evaluating the ecotoxicity and biodegradability of water-soluble polymers used in personal care products and cosmetics. Ecotoxicol. Environ. Saf..

[100] Li W.W., Li C.M., Gu Z.B., Qiu Y.J., Cheng L., Hong Y., Li Z.F. (2016). Relationship between structure and retrogradation properties of corn starch treated with 1,4-α-glucan branching enzyme. Food Hydrocolloid.

[101] Wu K., Li C., Li Z., Gu Z., Ban X., Hong Y., Cheng L., Kong H. (2024). Enzymatic modification lowers syneresis in corn starch gels during freeze-thaw cycles through 1,4-alpha-glucan branching enzyme. Int. J. Biol. Macromol..

[102] Buwalda P.L.D., Hofman A.M. (2020). Starch Based Aqeous Adhesive Compositons and Used Thereof. U.S. Patent.

[103] Mann K.J.K., Gruell M., Wastyn M.M.D. (2017). Starch-Based Glue Composition. U.S. Patent.

[104] Miao M., Bemiller J.N. (2024). Enzymatic approaches for structuring starch to improve functionality. Annu. Rev. Food Sci. Technol..

[105] Lang Y., Dong Y., Li X., Yan Q., Zeng G., Li J. (2026). Recent Progress on the Application of Hyperbranched Polymers in Biomass-Based Adhesives for Sustainable Wood Bonding. ChemSusChem.

[106] Chen Y.-F., Kaur L., Singh J. (2018). Chemical modification of starch. Starch in Food.

[107] Fan Y., Picchioni F. (2020). Modification of starch: A review on the application of “green” solvents and controlled functionalization. Carbohydr. Polym..

[108] Fan Y., Picchioni F. (2018). Controlled Synthesis of Starch-based Branched Polymers. Ph.D. Thesis.

[109] Choi K.O., Yim C.S., Nguyen P.C., Park J.T. (2025). Structural and functional variations of octenyl succinylated clustered amylopectin produced by distinctive glycogen branching enzymes. Food Sci. Biotechnol..

[110] Díaz de Los Ríos M., Hernández Ramos E. (2020). Determination of the Hansen solubility parameters and the Hansen sphere radius with the aid of the solver add-in of Microsoft Excel. SN Appl. Sci..

[111] Dona A.C., Pages G., Gilbert R.G., Kuchel P.W. (2010). Digestion of starch: In vivo and in vitro kinetic models used to characterise oligosaccharide or glucose release. Carbohydr. Polym..

